# B-cell maturation antigen targeting strategies in multiple myeloma treatment, advantages and disadvantages

**DOI:** 10.1186/s12967-022-03285-y

**Published:** 2022-02-10

**Authors:** Shirin Teymouri Nobari, Jafar Nouri Nojadeh, Mehdi Talebi

**Affiliations:** 1 Department of Medical Biochemistry, Faculty of Medicine, Urmia University of Medical Sciences, Urmia, Iran; 2 Department of Medical Genetics, Faculty of Medicine, Tabriz University of Medical Sciences, Tabriz, Iran; 3 Department of Applied Cells Sciences, Faculty of Advanced Medical Sciences, Tabriz University of Medical Sciences, Tabriz, Iran

**Keywords:** B-cell maturation antigen, CAR-T cells, Multiple myeloma, Therapy

## Abstract

B cell maturation antigen (BCMA), a transmembrane glycoprotein member of the tumor necrosis factor receptor superfamily 17 (TNFRSF17), highly expressed on the plasma cells of Multiple myeloma (MM) patients, as well as the normal population. BCMA is used as a biomarker for MM. Two members of the TNF superfamily proteins, including B-cell activating factor (BAFF) and A proliferation-inducing ligand (APRIL), are closely related to BCMA and play an important role in plasma cell survival and progression of MM. Despite the maximum specificity of the monoclonal antibody technologies, introducing the tumor-specific antigen(s) is not applicable for all malignancies, such as MM that there plenty of relatively specific antigens such as GPCR5D, MUC1, SLAMF7 and etc., but higher expression of BCMA on these cells in comparison with normal ones can be regarded as a relatively exclusive marker. Currently, different monoclonal antibody (mAb) technologies applied in anti-MM therapies such as daratuzumab, SAR650984, GSK2857916, and CAR-T cell therapies are some of these tools that are reviewed in the present manuscript. By the way, the structure, function, and signaling of the BCMA and related molecule(s) role in normal plasma cells and MM development, evaluated as well as the potential side effects of its targeting by different CAR-T cells generations. In conclusion, BCMA can be regarded as an ideal molecule to be targeted in immunotherapeutic methods, regarding lower potential systemic and local side effects.

## Introduction

**Fig. 1:**
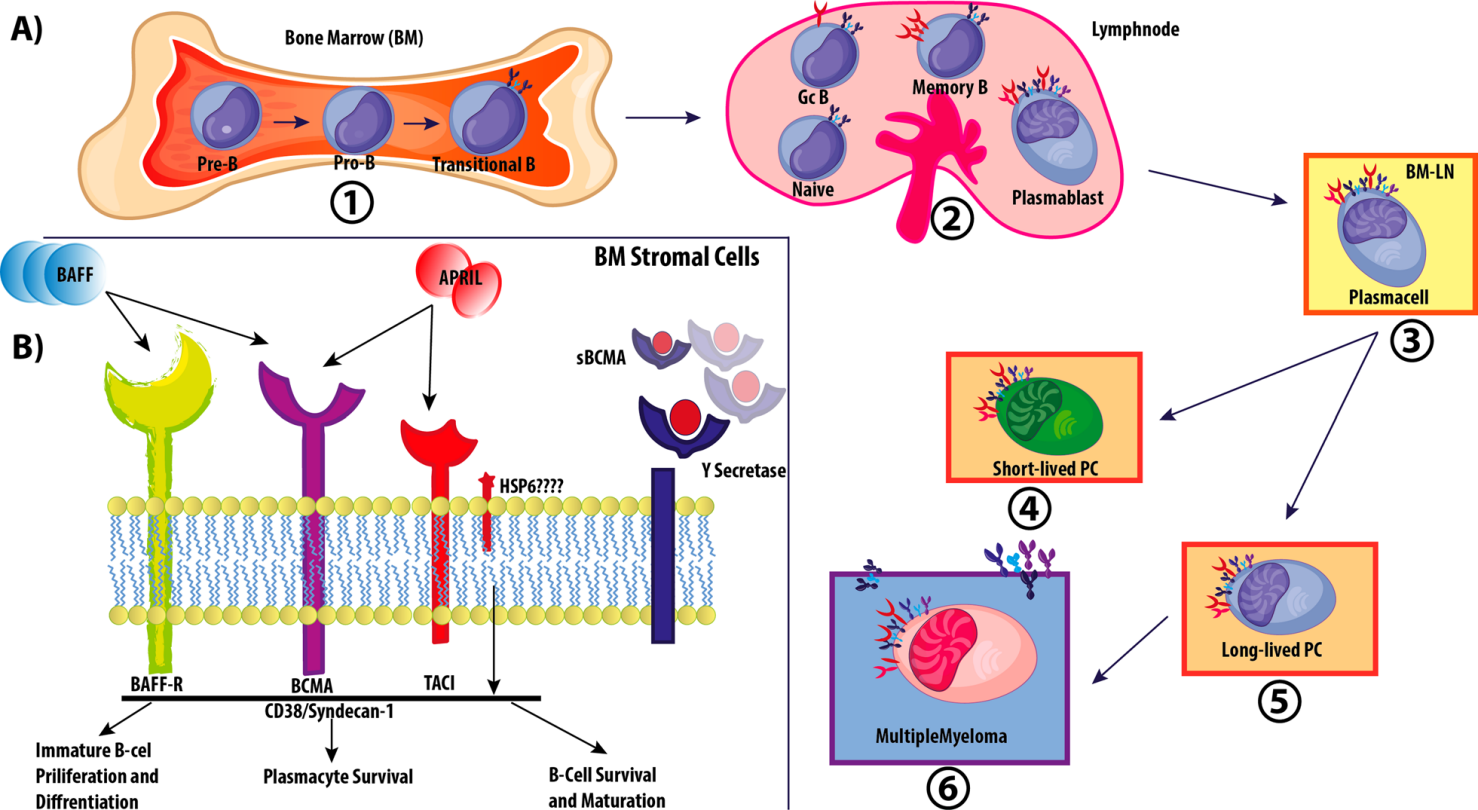
Expression of B cell maturation antigen on plasma cells. The stages of B cell differentiation take place in the bone marrow and Lymphnode. When memory cells differentiate into plasma cells, BCMA expression begins and is expressed on short-lived proliferating plasmablasts, and long-lived PCs, mature B-cells and malignant B cells which are much more pronounced in malignant cells. An example is multiple myeloma cells. BCMA isn’t critical for normal B-cell homeostasis but is required for the survival of long-lived PCs. induction of BCMA expression occurs with a BAFF-R decreasing during the differentiation of PCs. APRIL and BAFF are two ligands for BCMA, And BCMA has a closely related to calcium modulator and cyclophilin ligand interactor (TACI). In addition to binding to BCMA, these ligands bind to their receptors at the cell surface, triggering signaling pathways which promote the growth and survival of PCs and activate anti-apoptotic pathways. APRIL binds to sulfated side chains of heparin sulfate proteoglycan (HSPG) its binding site to bind to TACI and BCMA. APRIL/BCMA signaling pathway Increases the activity of malignant plasma cells. BCMA is converted to soluble BCMA (sBCMA) by the enzyme protease γ-secretase, sBCMA can interfere with signaling and the level of sBCMA is a marker for b cell involvement in some disease

Multiple myeloma (MM) is known as a malignancy of plasma cells (PCs) located in the bone marrow, that leads to excess production of abnormal immunoglobulins and bone destruction. MM is a primary malignancy of the BM PCs initiated by the transformation of memory B cells (CD19 + , CD 27 + , CD 38 + , CD45 − , and CD138 −) [1]. In recent decades, many therapy strategies have been developed based on monoclonal antibodies (mAb) (such as daratumumab or elotuzumab), proteasome inhibitors and immunomodulatory drugs. However, MM remains an incurable disease yet. Its severity and clinical and/or laboratory stages manifestations vary from a premalignant precursor, monoclonal gammopathy of undetermined significance (MGUS), to smoldering MM, and active MM finally [2]. The progression of multiple myeloma to invasive disease is due to genetic mutations and chromosomal abnormalities. Many of these alterations are associated with changes in metabolism, apoptosis, cell growth, and the epigenetics of MM cells [3]. MM cells are in close contact with BM accessory cells that eventually lead to the spread, survival, and escape of the immune system. These bone marrow stroma cells include endothelial cells, osteoclasts and osteoblasts, BM macrophages, regulatory T-cells (T_reg_s), plasmacytoid DCs (pDCs), dendritic cells, mesenchymal cells, and myeloid-derived suppressor cells. These cells support MM cells by producing a wide variety of cytokines, antiapoptotic and growth factors, for example, macrophage inflammatory protein-1α (MIP-1α), tumor growth factor β (TGFβ), B-cell activation factor (BAFF), A proliferation-inducing ligand (APRIL), and most importantly interleukin-6 (IL-6) [2] (Fig. 1). Important signaling pathways that are activated include STAT3, NF-κB, ERK1/2, AKT/PI3K, and play an important role in disease progression. New therapies directly target the growth and survival of MM cells which are necessary strategies in high-risk relapsed and refractory (RR) MMs [4]. B cell maturation antigen (BCMA) is the target of the choice antigen used in anti-MM immunotherapy. BCMA is a non–tyrosine kinase receptor surface glycoprotein that is widely expressed on malignant plasma cells and most MM cell lines as well [5]. BCMA by its ligand, APRIL, increases survival and long-lived plasma cells that contribute to MM development. It is closely related to the BAFF receptor (BAFF-R), that highly expresses on MM cells. The NF-κB pathway is mainly activated by binding APRIL or BAFF to BCMA and to protecting MM cells by activating anti-apoptotic proteins like; BCL-XL, BCL-2, MCL-1 [6–8]. TNF receptor activates BAFF on transcription, proliferation, survival, and differentiation of MM cells by activating NF-κB factor [9]. Chimeric antigen receptor (CAR) T or NK cells, GSK2857916 an antibody–drug conjugate, and bispecific antibodies are considered as several specific treatments for MM [10]. Through genetic engineering, T cells can detect cells that express BCMA. BCMA-specific CARs transfected T-cells, called anti-BCMA-CAR-T-cells demonstrated specific MM cells killing activity in vitro [11, 12]. Julia Bluhm et. al. [13] reported that BCMA can be an interesting target for CAR T-cells therapy approaches. Conventional treatments with monoclonal antibodies have lower side effects and costs than CAR-T cell but depend on the high concentration of BCMA expression in cells. Antibody–drug conjugates (ADCs) are strategies to increase mAb therapy. In this method, cytotoxic payload is directed to tumor cells that escaped from the immune system and bispecific mAbs bind T or NK cells to tumor cells, activating effective cells and lysing malignant cells [14].

Finding the Tumor-Specific Antigens as a unique marker for targeting tumor cells other than normal ones is the challenging part of any immunotherapy approaches, as in CAR-T cell therapy manipulating technics. There are some known relatively specific markers for tumoral plasma cells to distinguish from normal ones, such as CD38, CD138, G-protein Coupled Receptor 5D (GPRC5D) [15, 16] SLAMF7 (CD319), MUC1 (engineered CAR T Cells Targeting the Cancer-Associated Tn-Glycoform of the Membrane Mucin MUC1 Control Adenocarcinoma), as well as other non-specific markers such as CD44v6, CD56, NKG2, Lewis-X, but a higher and relatively specific expression of the BCMA on these cells, currently makes it an optimal but not ideal target in CAR-T cell therapy methods. During selecting process of the optimal immunologic target(s), Specific expression patern of the target is as important as the antigen shedding status of it, because soluble antigens participating in the mAbs neutralization or inactivates CAR T-cells, about this feature as the shedding process of BCMA is related to γ-secretase membrane enzyme function, controlling the shedding status is so easier than the other targeting options, theoretically. However, regarding the molecule expression pattern on the normal plasma cells, studying the full functional mechanisms, and local and/or systemic side effects of the targeting solely or in combination with other antigens, is the background aimed in this review, at the same time currently introduced mAb based approaches reviewed because of the vicinity of the both mAb and CAR T-cell technologies.

### BCMA structure and function

BCMA is a cell membrane type III non-tyrosine kinase receptor glycoprotein [17–19]. This protein does not have a signal peptide, its extracellular residues are rich in cysteine [20, 21]. There are six motifs in the N terminal section of this receptor, which indicates that the BCMA is a member of the tumor necrosis factor receptor superfamily 17 (TNFRSF17)/CD269 [2]. TNF and TNF receptors family members are important in enhancing immune functions [22]. It is specifically expressed on plasma blasts and plasma cells (PCs) [23]. It is detected in the interfollicular region of the germinal centers but no evidence of expression in the follicular mantle zone has been reported [24]. Lack of BCMA doesn’t affect the number of normal B cells but disrupts long-lived plasma cells [24, 25]. Firstly, Tsapis et. al. described the BCMA gene through molecular analysis of t(4;16)(q26; p13)/IL2/TNFRSF17 in human intestinal T-cell lymphoma [19]. BCMA was predicted to be an integral transmembrane protein with 24 hydrophobic central amino acids region in an α-helix structure [26], containing three exon regions separated by two introns that encode 185 amino acids peptide [18].

As mentioned, BCMA is a glycoprotein whose glycosylation is a common practice for modulating membrane proteins [27] and this process keeps the protein on the cell membrane [28]. The N-glycan site in BCMA is probably in the asparagine (N) residue at 42nd amino acid (N42). The N-glycosylation is important because of its role in regulating plasma cell function through ligand binding control. In addition, BCMA glycosylation, especially its sialylation, promotes cell survival [15, 25].

Recently, two members of the TNF superfamily proteins called B-cell activating factor (BAFF) and A proliferation-inducing ligand (APRIL), that BCMA closely interacts with, have been identified and their role in the maturation and differentiation of B cells have been described [16].

BAFF (BLyS, TALL-1), a member of the tumor necrosis factors superfamily, is known to stimulate B cells [29]. This molecule, which is mainly expressed by macrophages and dendritic cells, is the survival signal for peripheral B cells [30, 31]. In some B cell malignancies, such as myeloma and autoimmune diseases, increase BAFF expression has been shown [32, 33]. During the study of systemic lupus erythematosus (SLE), it was found that overexpression of transgenic BAFF caused autoimmune disease [33, 34] so that it may play a role in autoimmune disorders [35, 36]. In many B-cell neoplasms, BAFF signaling becomes inefficient and causes tumor cells to grow and survive by creating an autocrine ring [33, 37]. BAFF also promotes tumor cells by activating NF-κB (nuclear factor kappa- B), BCL2, BCLX(L) upregulation, and downregulation of BAX [38].

BAFF binds to three specific receptors on B cells: BAFF receptor, TACI (transmembrane activator calcium modulator and cyclophilin ligand interactor), and BCMA. It binds to BCMA in normal cells to increase cell survival, proliferation, differentiation, and antibody production [30, 39]. Serum levels increasing of BAFF shown in multiple myeloma patients [8, 40], but the BAFF receptor is difficult to detect on malignant plasma cells [41] and so suggesting that it has less effect on the survival of multiple myeloma cells [42].

APRIL was initially detected on tumor cells; it is secreted by myeloid cells and penetrates the bone marrow during abnormal myelopoiesis in multiple myeloma. It was later shown to be able to secrete immunoglobulins and class switching involved in B cells. Multiple myeloma cell line is dependent on interleukin-6. In the absence of this interleukin, APRIL protects cells [8, 29, 43] and saves them from dexamethasone-induced apoptosis [8]. APRIL binds only to BCMA and TACI [16], Binding to BCMA suppresses the immune system in the bone marrow and increases the growth of multiple myeloma cells. this physiological relationship indicates that BCMA has greater affinity and interaction with APRIL [44, 45]. APRIL promotes the survival of malignant plasma cells through heparan sulfate proteoglycans, which its roles in regulating cell adhesion, cytoskeletal re-organization, migration, and growth factor signaling have been shown [46–50]. This indicates that APRIL has a more specific role than BAFF [46]. Both BAFF and APRIL are involved in tumor cells by transmitting antitumor signals [51]. In patients with multiple myeloma, they increase compared to normal people[52]. BAFF and APRIL stimulate multiple myeloma cells through anti-apoptotic molecules such as BCL2, MCL1 [6, 29, 43].

TACI expressing on mature B cells upregulates on activated B cells and plasma cells [53]. In humans, TACI (TNFRSF13B) gene mutations in humans are shown in about 10% of patients with Common Variable Immuno-Deficiency (CVID) disorder, which manifests with impaired antibody production and are more susceptible to Streptococcus pneumoniae and Hemophilus influenzae infections, as well as autoimmune diseases [54, 55].

### BCMA expression

When BCMA was firstly cloned from human T cell lymphoma, noticed that its expression was associated with B cell maturation and the highest level observed in the plasma cell line [19]. BCMA protein is located in the Golgi apparatus, which its expression is relatively limited to a specific cell lineage, B cells, so it a hypothesis that as the Golgi apparatus is larger and more abundant in plasma cells, it may perform as an antibody secretion facilitator [28].

BCMA expression has been tracked on differentiated PCs a well as plasma blasts. This protein is produced in memory B cells differentiating to plasma cells and is present in all PCs but not in CD34 + HSCs, naive B cells, and other normal tissue cells [16, 25, 56–58]. Blimp-1(B-lymphocyte-induced maturation protein 1), a gene controlling the proliferation of PCs, has a positive inducer of BCMA expression [59].

Induction of BCMA expression occurs with a BAFF-R decreasing during the differentiation of PCs [25, 60]. BCMA is present on the surface of mature and malignant B lymphocytes too [19, 40, 61, 62], so its expression is not limited to normal cells and tissues [15]. BCMA membrane expression has been detected by anti-BCMA antibodies in CD138 + multiple myeloma cells [21], more commonly in malignant cells than in normal PCs and other bone marrow cells [63]. This observation is confirmed by multiple gene expression profiling and immunohistochemistry [21]. In a study by Friedman et al. MM cells and even primary MM cells show a strong expression of BCMA [64]. BCMA was detected using Chromatin immunoprecipitation, which is required for the analysis of IRF4, a transcription factor for MM [65], also its expression is preserved in MM patients after treatment [66]. Regulated and widespread expression of BCMA on MM cells stimulates cell growth and suppresses the immune system in the bone marrow [5]. In the Kinner et. al. study, primary bone marrow samples were taken from eighth patients with MM to analyze the expression of BCMA on the surface of MM cells and myeloma progenitor cells (MPC), MPCs do not have the plasma cell phenotype and are not completely differentiated [5], they have a weaker response in patients to treatments such as stem cell transplantation and proteasome inhibiting [67]. In several hematological tissues including bone marrow, tonsils and spleen, lymphnodes, white blood cells, BCMA isoforms were detected by qPCR [40], its expression in various blood cells, and Hodgkin lymphoma was assessed by flow cytometry [68], as well as in glioblastoma [69], chronic lymphocytic leukemia [70, 71], and Raji-Burkitt's Lymphoma and primary lymphoma [61, 72]. No expression could be detected in endothelial cells, keratinocytes, fat cells within tissues [73, 74] and in other blood cells including neutrophils, macrophages, and T cells [75, 76]. In addition, there is another type of PCs called plasmacytoid dendritic cells (pDCs) that is involved in the survival and drug resistance of MM cells [77]. These cells have significantly lower BCMA expression than PCs [78], pDCs located in the bone marrow near MM cells to enhance their growth and survival [77], so the role of BCMA in pDCs causes further enhancement of the viability and drug resistance of MM cells [77].

A study in the UK on 70 MM patients showed that BCMA expression was maintained through disease recurrence, extramedullary spread, and residual disease [66]. Tai et. al. showed that BCMA is expressed on the MM cells and is limited to plasma cells. The density of BCMA on the cell surface was measured using MFI (Mean Fluorescent Index) by flow cytometric analysis [63]. An enzyme called γ-secretase, a multi-subunit protease cleaves BCMA to release its soluble form called sBCMA [79]. The level of sBCMA is a marker for B cell involvement in known autoimmune diseases [80] and is more closely related to the patient's clinical condition [81]. In Systemic Lupus Erythematosus (SLE), the serum level of sBCMA is strongly associated with disease activity [82]. In a study of 209 patients on new case multiple myeloma, the level of sBCMA was significantly lower than in the control group and its significance in monoclonal gammopathy was not determined [63]. Also, in patients with indolent MM, the amount of sBCMA is less than active MM. In addition, the amount of this protein in MM disease is associated with clinical response, overall survival and is inversely related to the production of polyclonal antibodies in these patients [63]. In the studies of Germezi et. al. who introduced sBCMA as a biomarker that can control and predict the results of MM patients and by examining 243 patients, the level of this protein measured by ELISA method in smoldering MM and active MM was high, in addition, sBCMA levels are correlated with plasma cell ratio at biopsy, patient's clinical status, and M protein [25, 83, 84]. As a result, the study of BCMA expression could serve as a target for access to antitumor effects in MM patients [63].

### Role of BCMA in the signaling pathways

BCMA mainly plays an important role in B cells for their proliferation, survival and also differentiates them into plasma cells [17, 25]. Humoral immunity status is affected by BCMA probably via increasing the survival of normal plasma blasts and PCs [39, 85]. BCMA does not appear to be critical for overall B cell homeostasis as it is not presented in naïve and memory B cells, but for the survival of Long-lived PCs in the BM is necessary [25, 60]. BCMA-related factor, BAFF-R, acts as the main receptor for B cell survival. Another protein TACI plays a negative but important role in regulating B cell homeostasis and autoimmunity. Continuous expression of BCMA in multiple myeloma prototypes indicates that it is a receptor for regulating prosurvival pathways [68].

APRIL and BAFF, which are ligands of the TNF family, are associated with three members of the TNFR, including TACI (CD267, TNFRSF13B) [86], BAFFR (BR3, CD268, TNFRSF17) [87, 88] and BCMA (CD269, TNFRSF13C) [19]. The structure of glycosaminoglycans, such as those found in Sindcan1 (DC138), is the independent junction of APRIL and TACI [50, 89]. Figure 1 has summarized the process.

**Fig. 2:**
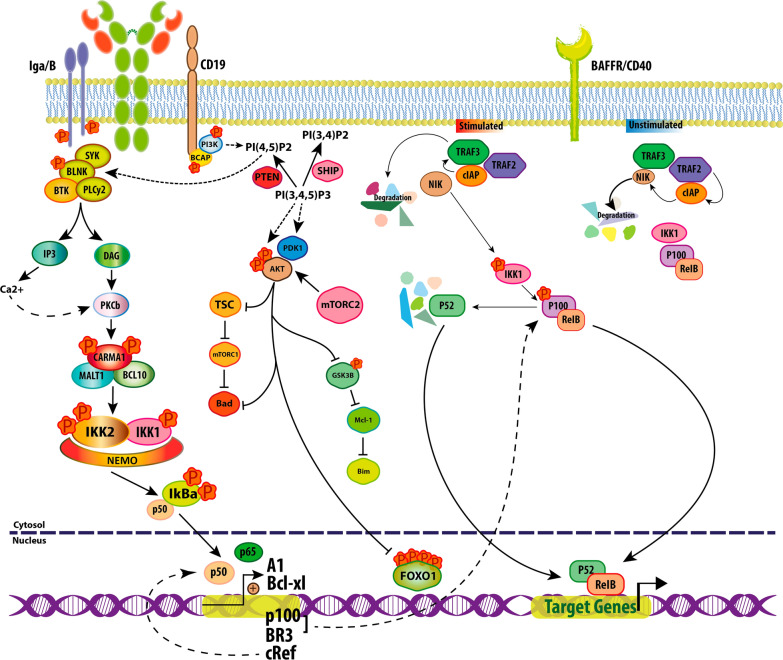
B-Cell Receptor (BCR), CD19 and CD40/BAFF receptors signaling, relationships and cross talk(s). The NF-κB is the most important pathway that activating by two: the classical (Canonical) and the alternative (noncanonical) pathways, with transcription factors including NF-κB1 (P50 and its precursor P105), NF-κB2(P52 and its precursor P100), RelA (P65) RelB, and c-Rel. The alternative pathway is the major pathway for B cell survival through BAFF-R, characterized by the presence of IKK1 and P100 phosphorylation cleaving to P52. The processed p52 heterodimerize with RelB, migrates to the nucleus, and induces transcription of anti-apoptotic genes. IKK1 is also phosphorylated by NIK. In unstimulated cells, TRAF3, TRAF2, and cIAPs1/2 factors are linked together, NIK is continuously destroyed by the proteasome, TRAF3, TRAF2, and cIAPs1/2 are factors for NIK ubiquitination and targeting it for degradation. After cell stimulation, TRAF3 is exposed to BAFF-R, which causes TRAF3 self-degradation by cIAPs 1/2 and TRAF2, this action leads to the stabilization of NIK and eventually causes cleavage of P100. The NF-κB alternative pathway is activated by the CD40 receptor too, a member of the TNF family. The BCR on mature naive recirculating B cells is achieved by the association of Igα/Igβ heterodimer. The classic pathway activated by the formation of P50 and P65 dimers after BAFF-R stimulation. Activation of canonical NF-κB signaling inducing through the Carma/Bcl10/ Malt1 (CBM) complex. In B cells, the PI3K signaling pathway activates PKCβ, so phosphorylated CARMA1 increases canonical activation of NF-κB through the CBM complex and phosphorylation of IKK2 by the TAB/TAK complex. IKK1 can contribute to the canonical IKK2/Nemo pathway, imparting important survival signals and it is also important in B cells for GC formation. recent studies show that the BCR induces p100 to facilitate BAFF-R signaling. The expression p100 acts as an inhibitor of p50 and p65. Therefore, canonical and non-canonical NF-κB pathways have special properties that ultimately determine the tempo and specificity of gene expression. Akt by disabling FOXO1, Prevents transcription of proapoptotic genes. It is observed that in the absence of FOXO1, peripheral B cells accumulate

There is a BAFF signal that is required for cell survival during differentiation, besides the BCR signal, that its downregulation results in the loss of more than 90% of mature B cells[90, 91]. As mentioned, TACI acts as a negative regulator in the maturation process of B cells, yet BCMA has no role in this stage whereas its role is in the later stages of differentiation [60, 92–94]. In a study of 293 transfected cells, it was observed that increasing the BCMA expression activates the NF-κB signaling pathway, relating to TRAF2, TRAF5, TRAF6, IKK1, and IKK2 elements [60, 95] (Fig. 2).

### BAFF-R signaling pathways

The APRIL-BAFF bonding role dominates in the next step of B-cell differentiation [96]. BAFF and its receptor play an important role in the development and survival of B cells [97]. Although BAFF does not induce cell proliferation alone, cells prepared with BAFF invitro transcribe the proteins required by the cell cycle, and BCR-induced proliferation occurs more rapidly. Cell size and protein content of the cells is positively controlled by BAFF, as well as forcing cells to glycolytic metabolism [98]. Elevated BAFF levels play a role in autoimmune diseases, so it is important to understand the supportive signaling pathways in B cell survival [97]. The NF-κB is the most important pathway that activating by two: the classical (Canonical) and the alternative (noncanonical) pathways, with transcription factors including NF-κB1(P50 and its precursor P105), NF-κB2(P52 and its precursor P100), RelA (P65) RelB, and c-Rel [99] (Fig. 2). The alternative pathway is the major pathway for B cell survival through BAFF-R, characterized by the presence of IKK1 and P100 phosphorylation cleaving to P52 [100]. The processed p52 heterodimerize with RelB, migrates to the nucleus, and induces transcription of anti-apoptotic genes. IKK1 is also phosphorylated by NIK [101]. In unstimulated cells, TRAF3, TRAF2, and cIAPs1/2 factors are linked together, NIK is continuously destroyed by the proteasome, These three sets(TRAF3, TRAF2, and cIAPs1/2) are a factor for NIK ubiquitination and targeting it for degradation[102, 103]. After cell stimulation, TRAF3 is exposed to BAFF-R, which causes TRAF3 self-degradation by cIAPs 1/2 and TRAF2, This action leads to the stabilization of NIK and eventually causes cleavage of P100 [103, 104]. The NF-κB alternative pathway is activated by the CD40 receptor too, a member of the TNF family (Fig. 2).

### BCR signaling

Signal transduction by the BCR on mature naive recirculating B cells is achieved by the association of Ig-α/Ig-β heterodimer. The classic pathway is activated by the formation of P50 and P65 dimers after BAFF-R stimulation [105]. Also, the activation of canonical NF-κB signaling is induced by the Carma/Bcl10/ Malt1 (CBM) complex. In B cells, the PI3K signaling pathway activates PKCβ, so phosphorylated CARMA1 increases canonical activation of NF-κB through the CBM complex as well as the phosphorylation of IKK2 by the TAB/TAK complex [106]. In addition, IKK1 can contribute to the canonical IKK2/Nemo pathway by giving some important survival signals [107, 108] and it is also important in B cells for GC formation (Fig. 2). Also, the BCR prompts p100 to facilitate BAFF-R signaling. The expression of p100 acts as an inhibitor of p50 and p65 [108]. Therefore, canonical and non-canonical NF-κB pathways have special properties that ultimately determine the tempo and specificity of gene expression [109].

### PI3K pathway

Another pathway downstream of BAFF-R is called PI3K, which plays an important role in BCR signaling and helps B cell survival. Recent studies showed that PI3K signaling induction correlates with B cells maturation defects improvements [98, 110, 111]. The class IA PI3Ks comprise of three catalytic isoforms (p110α, β, and δ) that form heterodimers with adapter subunits (p85α, p55α, p50α, p85β, and p55γ), whose functions are regulating enzymatic activity [112]. p110 can play its role by applying p85 with transmembrane adapter CD19 associated with cytosolic BCAP in B-cell receptor signaling. PtdIns(3,4,5)P3 and PtdIns(3,4)P2 are may be substrates for the phosphoinositide 3-phosphatase PTEN, which seemed like the main functional antagonist of PI3K [113]. Production of PtdIns P3 stimulates cell growth, proliferation, survival, and differentiation pathways. By Akt phosphorylation, BAFF induces PI3K activity [98] (Fig. 2). The significance of this induction is that cells in p110δ deficient have difficulty responding to BAFF-induced survival [114]. In regard to the downstream effector pathways, BAFF interaction with Btk, PKCβ, and Akt promotes ribosome biogenesis and enhances metabolic activity to prime B cells for antigen-induced proliferation [115, 116]. Also, BAFF increases the regulation of the pro-survival factor Mcl-1 by the Akt-dependent inactivation of GSK3α/β [117]. Akt by disabling Foxo1 prevents transcription of proapoptotic genes. It is observed that in the absence of FOXO1, peripheral B cells accumulate [118, 119]. PI3K binds to adapter proteins CD19 and BCAP and produces PtdIns P3, which in turn employs PLCγ2 and Btk. Btk activates PLCγ2, increases DAG production, and enhances intracellular Ca2 + release which merges to activate PKCβ. PKCβ activation is critical for the canonical NF-Κβ pathway.

It is possible that the activation of Mcl-1 expression is regulated primarily in a post-translational manner which needs PI3K signaling. Also, it should be noted that some of the BH3-only family members are inhibited by the PI3K family. For instance, Bad is destroyed via phosphorylation by Akt, Bim, and Puma and becomes the targets of FOXO factors [120, 121].

### CD40 receptor

CD40 is one of the main members of the TNF family that affects B cell biology [109]. CD40 expression occurs during B cell development, in the B cell transition phase, its signals support BAFF-R expression and possibly cell survival or homeostatic proliferation [122, 123]. The presence of CD40 on mature cells stimulates proliferation, in GC, supports B cell survival, differentiation, and isotype switching [124]. CD40 is vital for the initiating of T cell-dependent B cell activation and therefore plays an essential role in humoral immunity response [97, 125]. CD40 signaling is mainly activated through canonical and noncanonical NF-kB pathways, and other signaling pathways such as MAPK, PI3K, and PLCg approximately after CD40 engagement [126–128]. Stimulation of CD40 causes the uptake of TRAF proteins. In this proteins family, TRAF2, TRAF3, and TRAF6 can bind directly to the cytoplasmic tail of CD40 but are indirectly associated with TRAF1 and TRAF5 [129, 130]. TRAF6 activates TAK1 resulting in activation of the canonical NF-κB signaling pathway [131, 132]. TRAF2 with MEKK1 kinase activates Jnk and P38, which is important in response to CD40 ligation [128]. TRAF2 and TRAF3 with CD40 cause NIK accumulation and consequently activate the alternative NF-κB pathway [103].

### APRIL signaling

APRIL is expressed in a large number of tumors and stimulates cell growth [133]. For example, in myeloma cells, it activates the MAPK, PI3K⁄AKT, and NF-κB pathways, which leads to an up-regulation of Mcl-1 and Bcl-2 anti-apoptotic proteins [8]. Also, APRIL can bind to heparan sulfate (HS) [49, 50], by its lysine-rich region in the N-terminal portion. The APRIL TNF-like free region communicates with BCMA and TACI receptors [46]. TACI-Fc also binds to HS chains including syndecan-1 [89]. The role of syndecan-1 in interaction with cellular matrix proteins, chemokines, growth factors, and adhesion molecules has been identified [134]. A study by Je´roˆme Moreaux et. al. [46] showed that MM cells can bind to a considerable quantity of APRIL and soluble TACI via cell surface syndecan-1 which this binding to syndecan-1 is essential for APRIL myeloma cell growth and survival. Overexpression of BCMA stimulates APRIL and activates both NF-κB pathways. In addition, it increases angiogenesis, metastasis factors, and the expression of growth and survival genes [5]. One study found that APRIL was associated with the expression of VEGF, its receptor, and CD138, as well as with the progression of MM [135].

Several studies show that BAFF binding to BCMA or TACI induces different signaling pathways such as NF-κB, P38 mitogen-activated kinase for BCMA [95], NF-κB nuclear translocation, and Jun-N-terminal kinases (JNKs) phosphorylation for TACI [136]. Also, previous studies had shown that continuous expression of BCMA in T293 cells, activates pathways including mitogen-activated protein kinase (MAPK), especially JNK, P38 kinase, NF-κB, and Elk-1 without stimulation of BAFF or APRIL [95]. Recent findings suggest that in MM, functional mutations occur in both canonical and non-canonical NF-κB. These mutations cause the activation of a variety of molecules such as NFKB1, NFKB2, NIK, CD40, and TACI, and inactivation of TRAF2, TRAF3, cIAP1/cIAP2 as well. Inactivation of TRAF3 represents one of the most common mutations in MM [137, 138] which leads to irregularity and amplification of both NF-κB pathways through the continuous presence of NIK. In some cases, NIK expression is necessary for the proliferation and spread of MM [139].

### Therapy

MM is the second most common hematopoietic malignancy in which malignant neoplasms of plasma cells accumulate in the bone marrow [140, 141]. This malignancy is caused by changes in memory cells (CD19 + , CD 27 + , CD 38 + , CD45 − , and CD138 −) [1], causing the development of osteolytic bone lesions and excessive production of monoclonal immunoglobulins in the blood and urine [140, 142]. MM arises from a precursor malignant disorder called monoclonal gammopathy of unknown significance (MGUS) and then progresses to smoldering MM (SMM), then active MM, which can eventually lead to PC leukemia [143, 144]. BCMA expression gradually increases from the MGUS stage to more advanced stages of multiple myeloma, including SMM and active MM [21]. In recent decades, various therapies have been used as mAbs such as proteasome inhibitors (PI) (e.g., Bortezomib), immunomodulatory drugs (IMiDs), (e.g., lenalidomide, daratumumab and elotuzumab) [145]. The use of PI and IMiDs combinations improves the response, in addition to increasing the overall survival in recurrent MM patients. The mAbs, which are the immunotherapeutic approaches, also improves the outcome of the disease, but since drug-resistant clones are always emerging, the disease remains incurable for most patients, so continuous researches for new treatments are necessary [146–148]. These methods resulted in a better response and prolonged survival, that have been summarized in Table 1.

**Table 1:** Immunotherapy approaches in anti-myeloma treatments

Technology	Targeted molecule		Introduced drug	Mechanism of action	References
Mono-clonal antibody-based technologies	Anti-CD38		Daratumumab	ADCC, ADCP, CDC	[62]
			Isatoximab	ADCC, ADCP, CDC, Pro-apoptosis	[62]
	Anti-SLAMF7		Elotuzumab	ADCC via NK cell activation through EAT-2 and CD16	[93]
	Antibody–drug conjugates (ADCs)	Anti-BCMA	Belantamab mafodotin (GSK-2857916)	Humanized anti-BCMA IgG1 MoA conjugated to monomethyl auristatin F (MMAF)	[36, 37]
		Anti-CD138	Indatuximab ravtansine	Targeting CD138, linked with maytansinoid cytotoxic agent	[38]
		Anti-CD56	Lorvotuzumab-mertansine	Targeting CD56, linked to a microtubule inhibitor (MD1)	[40]
		Anti-CD74	Milatuzumab doxorubicin	Targeting the CD74 linked to doxorubicin	[8]
	Bispecific monoclonal antibodies (Bs mAbs)	CD19/CD3	Blinatumomab	Cytotoxicity induction by accumulating T-cells to CD19 + cells	[106]
		BCMA/CD3	AMG-420	Cytotoxicity induction by accumulating T-cells to BCMA + cells	[104]
		BCMA/CD3	AMG-701	Cytotoxicity induction by accumulating T-cells to BCMA + cells with extended serum half-life in compared with AMG-420	[108]
		BCMA/CD3	teclistamab (JNJ-64007957)	Direct Cytotoxicity induction by accumulating T-cells to BCMA + cells	[110]
		CD38/CD3	GBR-1342	Direct Cytotoxicity induction by accumulating T-cells to CD38 + cells	[98]
		CD38/CD3	AMG-424	Direct Cytotoxicity induction by accumulating T-cells to CD38 + cells	[104]
		FcRH5-CD3	Cevostamab-BFCR4350A	Direct Cytotoxicity induction by accumulating T-cells to FcRH5 expressing cells	[112]
		GPRC5D-CD3	talquetamab-JNJ-64407564	Direct Cytotoxicity induction by accumulating T-cells to GPRC5D presenting cells	[113]

Antibody-dependent cellular toxicity (ADCC), complement-dependent toxicity (CDC), antibody-dependent cellular phagocytosis (ADCP), Signaling lymphocytic molecule F7 (SLAMF7), B-Cell Maturation Antigen (BCMA), Fc Receptor H5 (FcH5), G-protein Receptor Coupled 5D (GPRC5D)

### Targeting BCMA with mAb in MM

The main function of the mAbs is to block growth factors signal transduction, cause growth arrest and apoptosis, or stimulation of deletion of mAb-coated target cells by activation of the host immune system by various Fcγ receptors(FcγR) expressed on the effector cells, calling Antibody-dependent Cell Cytotoxicity (ADCC) strategies [17]. Treatment with mAbs has a longer half-life than other anti-MM drugs in ongoing and completed clinical trials combining with lenalidomide/len and dexamethasone/dex with elotuzumab (elo) targeting CS1 (SLAMF7) [149], furthermore, daratuzumab (Dara) and SAR650984 (SAR) targeting CD38 [147, 150]. It should be noted that Dara and SAR exhibit clinical activity as monotherapy but, CS1 and CD38 are expressed in other hematopoietic cells that disrupt mAb utilization. IgG therapy helps to improve mAb function and is also used by antibody–drug conjugates (ADCs) to trap malfunctioning immune cells, and because MM patients have a recurrent immune system disorder, ADCs are needed to target specific antigens, directly and indirectly, to eliminate MM cells [17]. ADCs are one of the fastest-acting anticancer drugs whose function is to detect specific antigens on tumor cells, attach them, and then absorb a cytotoxic chemical (payload) along with their cargo to kill tumor cells [2]. Toxic consignments associated with ADCs include monomethyl auristatin F (MMAF), tubulin polymerization inhibitor, pyrrolobenzodiazepine (PBD), or the RNA polymerase II inhibitor, α-amanitin, applying a cleavable or non-cleavable linker [10, 78, 151]. Recently, an ADC was developed to target BCMA to kill MM cells with fewer side effects [78].

### J6M0-mcMMAF (GSK2857916)

J6M0 is a humanized anti-BCMA that competes with APRIL and BAFF for BCMA binding [17]. J6M0 is a mAb and IgG1 whose afucosylated state can bind to all MM cell lines due to its tendency to BCMA [78]. J6M0 has a stronger binding capacity to CD138 + cells than pDC cells, indicating an association between BCMA mRNA and its protein expression on cells. Because J6M0 with normal FC or afucosylation cannot directly lead to cell death, it is converted to J6M0 ADCs with the anticancer drug auristatin. J6M0 was linked to either valine-citrulline (vc; protease cleavable linker)-monomethyl auristatin E (MMAE) or maleimidocaproyl (mc; non-cleavable)-monomethyl auristatin F (MMAF) which uses these as cargo that has higher stability and anti-tumor function [2, 78, 152]. J6M0-mcMMAF (GSK2857916) binds more strongly to MM target cells and has no adverse negative impacts on BCMA-negative cells (NK, monocytes, PBMCs, or BMSCs) [17]. Afucosylated GSK2857916 continuously enhances antibody-dependent cellular cytotoxicity[78]. This mAb stops cell proliferation by blocking the cell cycle of G2/M and induces apoptosis by activating caspases 7, 3, and 8; moreover triggers ADCC and antibody-dependent cellular-mediated phagocytosis against patient MM cells [2]. This mAb was the first ADC therapy with three distinct MOAs (apoptosis, ADCC, ADCP) to eradicate MM cells in the BM microenvironment more effectively [17]. Recently Oca et. al. reported the maximum accumulation of GSK2857916 on tumor site in immune-competent mice injected with EL4 lymphoma tumors expressing human BCMA (El4-hBCMA) cells [153]. During Phase 1 dose-escalation and expansion handled by Trudel et. al. (NCT02064387) showed that at maximum dose of 3.4 mg/kg once every three weeks, in 60% of the patient partial response or better achieved [154], but based on Oca et. al. work, combination with other immune-check point therapies shows much better result that monotherapy once [153].

### Chimeric antigen receptor T-cells

**Fig. 3:**
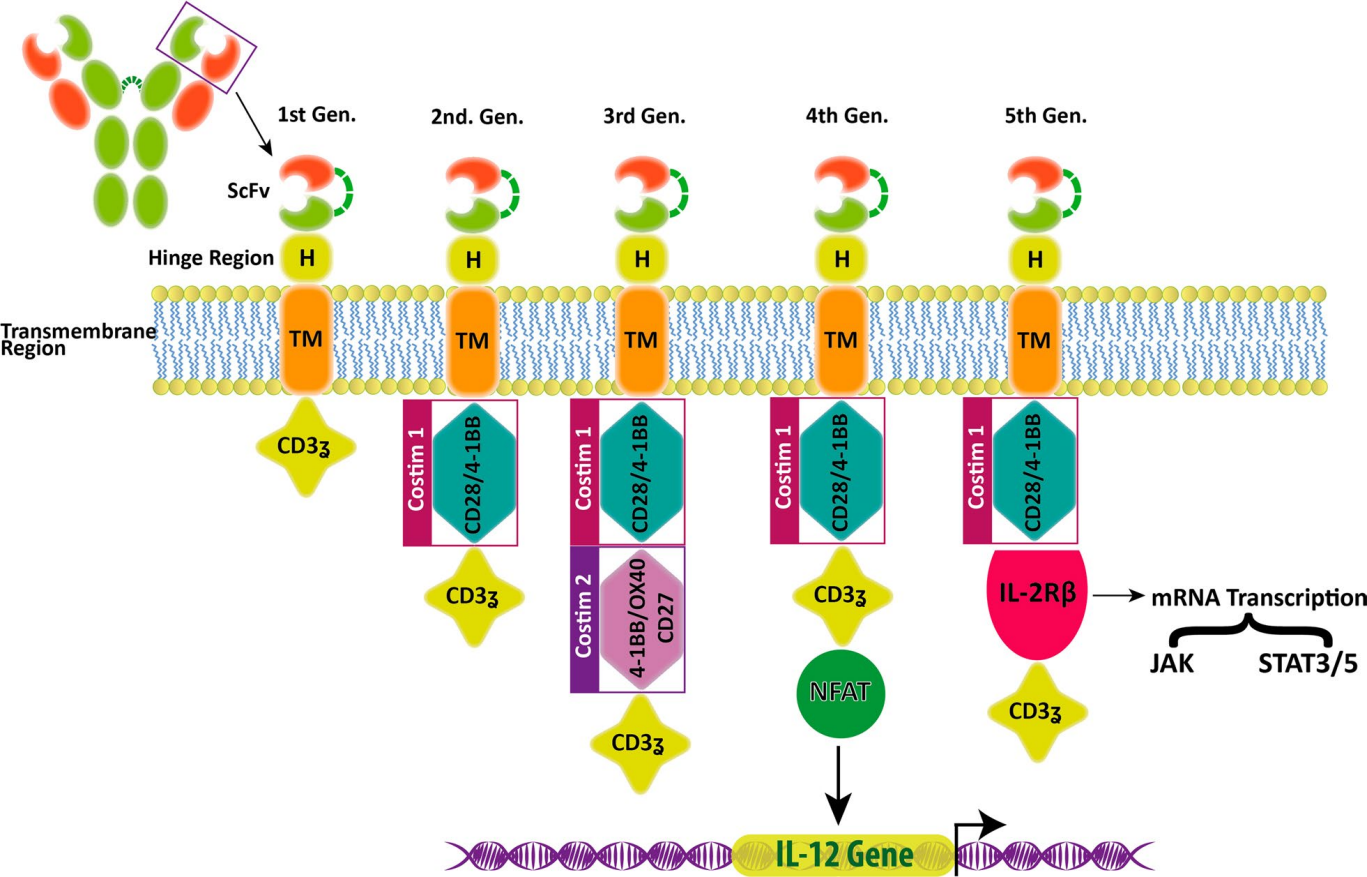
The types of CAR T cell. CAR is a hybrid receptor for antigen that is part of the antibody and part of the TCR and has two domains: extracellular antigen binding portion and an intracellular signaling domain. The extracellular domain includes the single-chain variable fragment (scFv), hinge with transmembrane domain (TM), and intracellular T cell activation domain of CD3ζ included three immunoreceptor tyrosine-based activation motifs (ITAMs). In the first-generation CAR T cell, we see a single structure of CD3ζ that acts as a signal transmitter from the endogenous T cell receptor that does not have enough power to activate the T cell and kill the target cell. CARs without costimulatory have no special function, so in the second generation costimulatory such as CD28 or 4-1BB were added to the cytoplasmic domain. This improves the proliferation and response process and increases the life of the CART cells. In the third generation, use a large number of signaling domains to produce potent cytokines with greater lethality. They equipped the fourth generation with nuclear factor activated T(NFAT) cells that stimulate cytokines such as interleukin 12. The fifth generation CARS contains IL-2Rβ, which activates the Janus kinases (JAKs) and signal transducer and transcription activator (STAT) signaling pathways. Function of costimulatory: CD28-mediated co-stimulation is important for regulating lymphocyte proliferation and survival. OX40 stimulates the production of interleukin 2. 4-1BB (CD137) plays an important role in maintaining T cell response signals and plays a major role in T cell survival and memory of CD8 + T cells

More recently, genetic therapy has been used in cell therapy approaches to manipulate T cell receptor genes to bind and kill tumor antigens [155]. Scientists have been introduced genetic engineering methods to produce chimeric antigen receptors (CARs) [156]. CARs are hybrid receptors for the antigen that is part of the antibody and part of the TCR and has an extracellular antigen-binding portion and an intracellular signaling domain [157]. The single-chain variable fragment (scFv) is derived from a tumor-specific antibody [158]. In mAb, the part that detects the antigen is integrated with CAR, which accompanies CD3ζ and a co-stimulatory molecule (such as intracellular activating domains of CD28 or 4-1BB) [159]. To achieve the final genetic construct for the CAR, a hinge and a transmembrane domain (TM), commonly from CD8 + cells or immunoglobulin bridge of the extracellular scFv and intracellular CD3ζ immunoreceptor tyrosine-based activation motif (ITAM) domains can be added to constructs [160] (Fig. 3).

The first generation of in vitro CARs possessed an intracellular signaling domain and consist only of CD3ζ to protect T-cell activation and target killing but, these CAR T cells had very limited persistence and antitumor efficacy in vivo. As a result, second-generation CARs were replaced to improve T-cell performance. TCR is for the detection of foreign peptide antigens that contain 8–12 amino acids [161], therefore, it may react with peptides that have similar sequences. Due to this, T cells need at least two signals to be fully activated. The first signal is provided by TCR and the second signal, or co-stimulation, is mediated through ligation of CD28 by CD80 or CD86, which are normally expressed on antigen-presenting cells (APC). CD80 and CD86 promote both signals and fully support T-cell activation, target killing, and long-term persistence. Therefore, T-cell activation fails when a T cell is exposed to a normal peptide on a normal cell [161, 162]. The scientists replaced the two-signal model of T-cell activation via modifying CARs to insert a CD28 costimulatory domain in tandem with CD3ζ ITAM domains [163, 164]. These second-generation CARs, their most important function, cause T-cell persistence and the elimination of effective tumors in vivo [165–167]. Second-generation CAR T cells have been proved to mediate strong anti-leukemia responses in clinical trials. Also, there is a third-generation CAR that includes CD28 and OX40 co-stimulation which stimulates the superior survival of CCR7 (−) T cells [164]. This CAR has less stimulation than IL-10 secretion compared to a second-generation CAR [168]. Fourth-generation CAR T cells, also commonly referred to as "TRUCK" T cells are produced to incorporate a third stimulatory signal [169]. They contain a nuclear factor of activated T cells (NFAT) domain, which induces a large number of cytokines (e.g., IL-12). This generation is equipped with immune-stimulating cytokines to improve the persistence of CAR T cells in a tumor environment that suppresses the immune system [170]. In addition, transgenic cytokine expression such as IL-12 can stimulate bystander T cells to kill antigen-negative cancer cells [169]. The fifth generation of CARs which have a fragment of the IL-2β (IL-2Rβ) receptor instead of the OX-40 / CD27 is being tested. Part IL-2Rβ induces the producing of Janus kinases (JAKs) and signal transducer and transcription activator (STAT) -3/5 [171, 172]. The problem with this new method is that, first, to detect tumor antigen by T cells, it is necessary to supply that antigen by antigen-presenting cells (APC), which is not possible in tumor cells. Secondly, T cells only detect tumor peptide antigens and are unable to detect antigens of polysaccharides, lipids, etc. that are present on the surface of tumor cells. Of the advantages of this method are, firstly, it is not necessary to present antigen by HLA molecules on the surface of APCs to detect tumor antigen. Second, since the binding site for CAR antigens is derived from antibodies, tumor cells antigens that reduce their HLA molecules to escape the immune system on their surface are also identified by CAR T-Cells [173].

### Treatment of multiple myeloma with CAR-T cells

The BCMA antigen is common and variable in all MM, and its expression is 25 to 100% in malignant plasma cells. A set of completely human BCMA-binding scFVs has been introduced by Bu et al. and has shown that this BCMA-specific antigen is commonly recurrent and resistant to treatment in phase I patients with multiple myeloma [65]. These chimeric receptors are transduced into the autologous T cell taken from the patient, by a retroviral or lentiviral vector or, more recently, by the Crisper/CAS9 method (for targeted placement within the genome and to prevent T cell tumor). And thereafter, new chimeric receptors are expressed on the cell surface. These T cells that express the chimeric receptors are called CAR T-Cells [156]. CAR T cells have high affinity and specificity to tumor cells as well as high cytotoxicity potential and proliferation [174]. In multiple myeloma, BCMA is the target antigen of choice commonly used in clinical trials of CAR-T cells [175, 176]. CAR T cells are also effective in treating acute and chronic leukemia and B lymphoma cells, where CD19 antigen is widely expressed. In MM, it has recently been reported that targeting activated integrin β7 can selectively eradicate MM cells including CD19 + clonotypic B cells [176–178]. Recently, a cancer-specific glyco-epitope called the Muc1 protein (Tn-Muc1) was shown as a suitable target for CAR T cells against a variety of cancers [179]. Therefore, to find mAbs that bind to MM cells, an antibody called MMG49 was identified, which binds to the integrin β7 protein, which, of course, binds only to the active integrin β7, thus MMG49 can play as a therapeutic target for removing MM clones [180]. Also, anti-MM CAR T cell therapy targeting BCMA has been tested in phase I clinical trials, and promising results were recently obtained from NCI's group [179, 181]. In a clinical trial conducted by Ji Xu et al. in 2019, targeting CAR against BCMA antigen in 17 patients with multiple myeloma (RRMM) after lymphatic chemotherapy has shown promising results and the overall response rate was 88.2% [182]. Besides relatively higher efficiency of the method, some limitation of CAR-T cell therapy needs to be overcome, basically therapeutic resistance grossly as result of tumor heterogeneity and antigen escape, and toxicity mostly because of cytokine releasing syndrome (CRS), and neurotoxicity mediated by pro-inflammatory cytokines following manipulated T-cells activation are most common disadvantages of the methods. Currently more than 100 clinical trials submitted for multiple myeloma targeting, that about 9 studies ended or nearly ending by results.

### Targeting membrane molecules other than BCMA

During conventional diagnosing protocols immunophenotyping studies of CD38/CD138 expression on suspected cells is the one of the key features for differentiating MM form other plasma cells dyscrasies or proliferations. So, it seems rational to search anti-CD38 and anti-CD138 as relatively specific tools targeting MM cells. CD38 expression level is constant during the disease stages but CD138 expression elevates during refractory and progressive stages [30–33]. Thus, these antigens seem are specific for MM but they express on other tissues, for instance CD138 express on normal tissues of hepatocytes, gastrointestinal goblet and columnar cells and squamous epithelium, at the same time, CD38 expresses on hematopoietic cells, Purkinje cells and lung smooth muscle cells. SLAM family member protein 7 (SLAMF7) expressing on normal T-cells, B-cells and NK-cells that targeting with mAb like elotuzumab, showed lysing of these SLAMF7 + normal cells too. G-protein coupled receptor 5D (GPRCP5D) expressing on the myeloma cells at high levels, so it can be regarded as a potent target in anti-myeloma immunotherapy strategies, but its expression on the normal plasma cells or mature B-cells in lower levels, as well as hair follicles questionable its specificity. Mucin 1 (MUC1) expressing aberrantly on MM cells, its intracellular domain interacts with β-catenin and serves as substrate for glycogen synthesis kinase 3β (GSK3β) that blocks β-catenin degradation, and so increasing the cells growth and proliferation by WNT/β-catenin. The MUC1 expression can be seen in solid tumors such as breast and colon carcinoma as well as numerous normal tissues such as, respiratory system, gastro-intestinal tract, kidney and urinary tract, female reproductive tissue and etc. that make it concerning its usefulness as specific multiple myeloma marker, despite its higher expression levels on MM cells.

## Conclusion

During choosing the most appropriate surface markers as specific tumor antigen, there are some key properties that should be taken account such as specificity to tumor cells not normal ones, higher and constant expression of the antigen and the shedding status of the antigen should be regarded. Among the variety of surface antigens that prone to consider as specific markers BCMA seem to more potent to be targeted but more shedding and growing BCMA-negatvie MM cells, that can cause escaping the tumor cells from immunotherapy strategies, should be considered and looking after a method that maximizing the targeting effectiveness from the beginning of immune cell therapy technologies is essential. So the maximum effectiveness of the CAR T-cell and other immunotherapeutic approaches is existing and expressing Cancer-Specific Antigen of tumor cell that differentiates these cells from normal ones in the same tissue, but in some cancers, there is no known cancer-specific antigens have been defined so, the most recent advances in CAR receptor designing by regarding “AND”, “OR”, “NOT” conditional functions, let the researchers produce more cancer-specific CAR T-Cells especially in the situations that there is no known cancer-specific antigen have been introduced.

## Data Availability

Not applicable.
